# Erector spinae plane block as an anesthesia technique for an emergent thoracotomy; a case report

**DOI:** 10.1186/s12871-024-02431-x

**Published:** 2024-02-08

**Authors:** Alireza Shakeri, Elham Memary

**Affiliations:** 1 Anesthesiology Department, Imam Hosein Hospital, Shahid Beheshti University of Medical Sciences, Tehran, Iran; 2 Anesthesiology Research Center, Shahid Beheshti University of Medical Sciences, Tehran, Iran

**Keywords:** Erector spinae plane block, Regional block, Regional anesthesia, Tamponade

## Abstract

**Background:**

The erector spinae plane block (ESPB) is a novel regional block technique for pain management following thoracic surgeries. However, there are minimal cases in which the technique was used as the main anesthesia technique during surgery.

**Case presentation:**

Here, we report the successful use of ESBP for applying anesthesia in a case during an emergent thoracotomy for performing pericardiotomy and loculated tamponade evacuation.

**Conclusions:**

Using ESPB with a higher concentration of local anesthetics, in this case, prepared appropriate anesthesia for performing an emergent thoracotomy while avoiding multiple needle insertions and the risk of further hemodynamic instability.

## Background

The erector Spinae plane block (ESPB) is a novel regional block technique primarily introduced by Forero et al. in 2016 for managing severe neuropathic pain [1]. Since then, ESBP has been mainly used for pain management following thoracic surgeries [2]. However, there are minimal cases in which the technique was used as the main anesthesia technique during surgery. Here, we report the successful use of ESBP for applying anesthesia in a case during an emergent thoracotomy. A written informed consent was obtained from the patient to publish identifying information.

## Case presentation

A patient in their 70s was first presented to the emergency department (ED) with complaints of orthopnea, leg edema, and melena. Her symptoms started several months ago and have progressed significantly during the last three days. She had a history of heart failure and underwent open surgical valve replacement for both mitral and aortic valves; for which, she had been taking various medications, including Warfarin. On review of the tests, the international normalized ratio (INR) was far above the normal range, and the hemoglobin (Hb) level was 3.8 mg/dl. On arrival to the ED, blood pressure (BP) was 100/50mmHg, and heart rate (HR) was 70/min. The patient was awake and alert; however, she was pale.

Cardiac monitoring and continuous pulse oximetry had been applied. A bedside echocardiography was performed which revealed cardiomegaly and mild to moderate pericardial effusion. With a provisional diagnosis of decompensated heart failure and gastrointestinal bleeding, the patient commenced on packed red blood cells (PRBCs) and fresh frozen plasma (FFP). The next day, the patient underwent both endoscopy and colonoscopy for approaching gastrointestinal bleeding.

Two days later, the patient’s dyspnea was increased, and in physical examination, an enlarged jugular venous pressure (JVP) was noted. The patient’s vital signs were BP = 95/45 mmHg, HR = 110/min, RR = 24/min, and SPO2 = 90% with a face mask. Emergency bedside echocardiography showed tamponade, for which pericardiocentesis was unsuccessful. According to the patient’s condition, history of previous heart surgery, high probability of the presence of pericardial adhesion, and loculated pericardial effusion, the patient was a candidate for an emergent thoracotomy. Therefore, she was transferred to the operating room (OR).

From admission to the ER until transferring to the OR, the patient received ten PRBC units and eight FFP units which makes Hb level to 8.9 mg/dL and INR to 1.26. In the OR, the patient was awake. However, she could not tolerate the supine position and refused general anesthesia. The patient’s oxygenation had been supported by a flow of 5–6 L/min of 100% oxygen via a face mask. After obtaining consent from the patient and coordinating with the surgeon, it was decided to perform ESPB on the left side via sitting position. Standard monitoring was established in addition to an arterial line obtained under local anesthesia. The tip of the scapula was identified as T7 and ultrasound-guided ESPB with an in-plane approach and high frequency linear transducer (Sonosite Edge II ultrasound machine 6–13 MHz) was performed by injecting 20 ml of Ropivacaine 0.5% in the fascia between the erector spinae muscle and the transverse processes of the 5th thoracic vertebrae. Then, 20 mg of intravenous ketamine was administrated. After 20 min, the thoracotomy incision site was checked by the pinprick test after ensuring sufficient analgesia, the patient was placed in the semi-lateral semi-sitting position. Limited left-side thoracotomy via 5th and 6th intercostal space between midaxillary and midclavicular lines with a length of 10 cm was performed. After that, a pericardiectomy was conducted, 200 mL of loculated serous fluid was removed, a water seal drainage was embedded, and the chest wall was sutured again. In all stages, the patient had complete anesthesia, and her hemodynamics were stable.

If, for any reason, the ESBP failed, plan B was to put the patient under general anesthesia, maintaining spontaneous breathing and avoiding positive pressure ventilation (PPV) until the removal of tamponade. Ketamine was intended as an induction agent. In case of insufficient spontaneous breathing, the plan was to use low volumes of mechanical ventilation. Inotropes and vasopressors were also available. Anesthesia was supposed to be maintained with an anesthetic balance technique using inhaled anesthetics and short-acting narcotics.

The patient was kept under observation for a while and then transferred to the cardiac surgery intensive care unit (ICU) to ensure proper recovery. The patient received no analgesic agent during the first 6 h post-surgery.

The drainage was removed on the 7th day of ICU admission, and the patient was transferred to the general ward. After 23 days of hospitalization, the patient was discharged with a satisfactory recovery, and no further complications were noted.

## Discussion

The first choice of anesthesia for thoracotomy is almost always general anesthesia [3, 4]. However, the patient neither tolerated nor consented to that. The epidural, paravertebral, or intercostal nerve blocks are generally used for perioperative analgesia or to reduce the dosage of anesthetics [5, 6]. However, an altered INR is the most crucial contraindication for a central block. In addition, in the epidural technique, blocking the sympathetic chain may alter the patient’s hemodynamic, however, this was contraindicated due to her unstable presentation upon arrival to the OR.

Hence, peripheral blocks, including paravertebral block, serratus plane block (SPB), and ESPB, may be favored options in these conditions. In the paravertebral approach, albeit lower than the epidural, there is still a chance of distributing to the epidural area and causing further hemodynamic instability [7]. Intercostal nerve blocks needed multiple needling [8], which may not be a good choice in this anxious patient who had an altered INR and coagulopathy. A systematic review and meta-analysis by KOO et al. revealed that although ESPB is less efficient than paravertebral block, it is preferable to SPB [9].

We decided to apply ESPB as a peripheral block approach, which was performed by just one needle insertion far from the epidural space. Meanwhile, a higher concentration of local anesthetic was administered to achieve proper anesthesia at the surgery site.

ESPB is a regional analgesia technique that targets the dorsal and ventral rami of spinal nerve roots. It is a less invasive option due to the avoidance of the neuraxial and paravertebral spaces. Furthermore, it is a safe technique used in various surgical interventions [6, 10]. 

ESPB has been extensively used in perioperative pain management for adults and pediatric patients undergoing thoracotomy [11, 12]. Intermittent or continuous ESPB was also conducted [13, 14]. The evidence revealed that applying ESPB leads to decreasing intraoperative and postoperative opioid use [15]. However, ESBP is not a routine anesthetic technique and has been rarely reported to be used in this regard [16–20]. This case report presents a successful course of applying ESBP for conducting thoracotomy.

## Conclusion

Using ESPB with a higher concentration of local anesthetics, in this case, prepared appropriate anesthesia for performing an emergency thoracotomy while avoiding multiple needle insertions and the risk of further hemodynamic instability.

## Data Availability

All data related to this case report are contained within the manuscript.
